# A Comparison of Military and Law Enforcement Body Armour

**DOI:** 10.3390/ijerph15020339

**Published:** 2018-02-14

**Authors:** Robin Orr, Ben Schram, Rodney Pope

**Affiliations:** 1 Tactical Research Unit—Bond Institute of Health and Sport; Gold Coast 4226, Australia; bschram@bond.edu.au (B.S.); rpope@csu.edu.au (R.P.); 2 School of Community Health, Charles Sturt University, Albury-Wodonga 2640, Australia

**Keywords:** police, armor, occupational tasks, load, personal protective equipment, army, defense

## Abstract

Law-enforcement officers increasingly wear body armour for protection; wearing body armour is common practice in military populations. Law-enforcement and military occupational demands are vastly different and military-styled body armour may not be suitable for law-enforcement. This study investigated differences between selected military body armour (MBA: 6.4 kg) and law-enforcement body armour (LEBA: 2.1 kg) in impacts on postural sway, vertical jump, agility, a functional movement screen (FMS), task simulations (vehicle exit; victim recovery), and subjective measures. Ten volunteer police officers (six females, four males) were randomly allocated to one of the designs on each of two days. Body armour type did not significantly affect postural sway, vertical jump, vehicle exit and 5 m sprint times, or victim recovery times. Both armour types increased sway velocity and sway-path length in the final five seconds compared to the first 5 s of a balance task. The MBA was associated with significantly slower times to complete the agility task, poorer FMS total scores, and poorer subjective ratings of performance and comfort. The LEBA was perceived as more comfortable and received more positive performance ratings during the agility test and task simulations. The impacts of MBA and LEBA differed significantly and they should not be considered interchangeable.

## 1. Introduction

Body armour is known to be effective in reducing fatalities in military environments [1]. However, body armour is no longer used solely by military populations, and the use of body armour is becoming more widespread among law-enforcement officers (LEOs) [2]. For example, one police force in New Zealand is citing the escalation of violent crimes as evidence of the need to regularly equip LEOs with body armour, particularly for protection against stab threats [2]. Given that a recent review of LEO injuries indicated that dealing with non-compliant offenders and assaults on officers was the leading mechanism of injury [3], this increased use of body armour appears warranted.

Unlike the military environment, which can see personnel rotate through deployment cycles and carry very heavy loads for intermittent periods (e.g., on patrol), the law-enforcement environment involves daily duties that span the officer’s occupational career and yet are ever-changing from minute to minute, day to day, and shift to shift [4]. Every year, police officers are exposed to risk as street-level bureaucrats handling law-enforcement, violent situations, negative attitudes, and threats from citizens [5]. Law-enforcement officers tasks can range from driving a patrol vehicle to checking an individual’s bona fides and attending domestic violence incidents [6]. On this basis, body amour may increasingly be a more constant, daily requirement for many contemporary LEOs, with limited periods of relief.

Noting that the work environment of LEOs is notably different from that of military personnel, it stands to reason that body armour requirements would be different, and a simple repurposing of any given military-purpose body armour may not be appropriate in a law-enforcement environment. As these police departments increasingly require officers to wear body armour, and procure body armour for them, it is important to consider factors such as relative performance and comfort in addition to the protections they afford. Uncomfortable or performance impairing body armour which officers perceive to provide inferior levels of protection will not be well tolerated and may be removed, thus losing its protective value. On this basis, the aims of this research project were to determine whether: (a) the physical movement capabilities of police officers differed when they were wearing current issue military body armour (MBA) as opposed to a specifically designed law enforcement body armour (LEBA); and (b) whether officers perceived there to be any differences in their ability to perform tasks or in their general comfort when wearing the two different types of body armour.

## 2. Materials and Methods

A prospective, within-subjects, repeated measures, randomised cross-over study design was used to address the study’s aims. Officers were allocated one of two light armour vests (Military Body Armour (MBA) or Law Enforcement Body Armour (LEBA)) using a randomised, counterbalanced approach. Officers wore their allocated vest (MBA or LEBA) for the duration of the first, single-day testing period and changed to the alternative vest for a corresponding test period on the second day. This study design meant that each of the officers acted as their own control while also controlling for any effects of learning by having half of participants using each of the two vests on Day 1 and then reversing these allocations on Day 2. In this way, if learning effects derived from Day 1 influenced Day 2 results, the impact of this learning would equally affect results for each type of vest, giving neither an unfair advantage.

All participants (females *n* = 6, males *n* = 4) were serving Australian law enforcement officers. Inclusion criteria for participants were that they: (a) had to be a current serving law enforcement officer; and (b) had no musculoskeletal injury or impairment that may affect any of the tasks. All officers volunteered to participate in the study while they were off-duty, and each participant provided written informed consent to participate prior to any testing. Ethics approval was obtained by the University Human Research Ethics Committee (protocol number 15803). The demographics of the participants can be seen in Table 1.

Throughout each testing period, officers were required to complete a series of measures and tasks that were chosen to address the aims of the research and because they were consistent with some of the activities LEO are expected to undertake on a day-to-day basis (Table 2). The testing periods were standardised across both days to mitigate the effects of potential diurnal variations.

Participants were weighed prior to their body amour being fitted, first in station wear (or equivalent), and then immediately after their allocated body armour had been fitted. The different body armour types were weighed for each individual, as they were expected to vary in weight based on sizing required. All weights were measured on a digital scale (Wedderburn WM204 Professional Weight Scale, Sydney, Australia). In addition, the height of all participants was measured (Ecomed Seca Measuring Rod, Hamburg, Germany) immediately after initial body weight measurements had been taken and prior to the participants donning their body armour. Participants were dressed in station wear (or equivalent), on which body armour was superimposed, in order to allow for a more accurate determination of the impacts of the different body armour types when superimposed (as is common practice) on standard work attire.

Each participant’s level of postural sway was measured with a force platform (AMTI Force Platform, Watertown, NY, USA), using AMTI Net Force Software (Watertown, NY, USA). Any increases in sway velocity may increase the risk of injury through impacting on recovery potential following a trip, slip or fall, a key cause of injury mechanism in police populations [7]. Participants stood with their feet together in the middle of a force plate with their eyes open, while looking straight ahead and standing as still as possible for 30 s. Variables of interest were measures of total sway (mm), average sway velocity (mm/s), average medio-lateral and anterior-posterior sway velocity (mm/s), and total excursion area (mm^2^). The postural sway data were analysed with AMTI BIO Analysis (Watertown, NY, USA) to derive each of these measurements.

Lower limb power output was measured using a vertical jump performed on a force platform (AMTI Force Platform, Watertown, NY, USA). Participants were instructed to take a step on to the platform before jumping up as high as possible, with a two-foot take off and using a natural arm swing. Participants were allowed a practice jump prior to delivering their best effort. AMTI Net force was used for data capture while AMTI BIO Analysis was used to derive the power output in Newtons (N). The vertical jump is a valid measure of leg power [8], and vertical jump scores have been associated with injury and illness risk in a police population [9].

The Illinois Agility Test was used to investigate the impacts of the different types of body armour on officer agility. Participants were instructed to lie in a prone position behind a start line, with their fingers placed beneath their foreheads. On the verbal command, ‘Go’, officers got to their feet as quickly as possible and ran around the premarked course which is 10 m long and 5 m wide and comprised of a mixture of straight line running, change of direction and weaving. Course time was measured by light gates (Fusion Sport, Brisbane, Queensland, Australia) and the time was recorded to the nearest 1/10 of a second. The Illinois Agility Test has been shown to be a valid measure of agility which assesses movement in different planes [10] and agility has been shown to be associated with performing key tasks in police officers [11].

A rapid car exit (General Motors Holden, Cruz Station Wagon, Adelaide, Australia) and sprint activity was used to replicate a scenario where officers were required to exit their vehicle rapidly in order to pursue an assailant or seek cover. The time to complete the task was measured using light gates (Fusion Sport, Brisbane, Queensland, Australia) and, following a self-determined medium pace practice, participants were required to complete the task as quickly as possible. At the commencement of this task, officers were seated in the driver’s seat of a vehicle without a seatbelt on, both hands were placed on the steering wheel, and the seat was adjusted to their specifications. The verbal commands ‘Ready’ and ‘Go’ were given by the researcher to start the scenario, with the light gate beam broken by the researcher’s hand on the latter command. The officer then exited the driver’s side of the vehicle and ran 5 m rearward, as quickly as possible. The 5 m rearward run distance was measured using a digital mini-measuring wheel (Senshin Industry Co., Ltd., Osaka, Japan). Time was recorded in seconds.

A victim recovery scenario was set up, using a load of 40 kg (2 × 20 kg plate weights), which was tethered via a gym belt to a 3 m hand loop. This task was designed to mimic retrieving a victim from an exposed area and dragging them back and behind cover. The task required participants to sprint 10 m from a start point toward the simulated victim (plate weights) before dragging them back along the same 10 m course. All times were recorded using a light-beam SMARTSPEED timing gate system (Fusion Sport, Brisbane, Queensland, Australia). Participants were permitted an initial practice run at a self-determined medium pace to familiarize themselves with the scenario and for warm-up purposes. Time was recorded to the nearest second.

To determine the officer’s mobility and movement quality while wearing each of the vests, a functional movement screen (FMS) was employed. The FMS assesses seven movement patterns, which include overhead squat, hurdle step, in-line lunge, shoulder mobility, active straight leg raise, push-up, and rotary stability [12]. Each component of the FMS is scored on a scale of zero to three points. A score of zero is assigned if the participant experiences pain with any portion of the movement pattern, a score of one indicates that the participant does not experience pain but cannot complete the movement pattern as instructed, and a score of two identifies that the participant can complete the movement pattern pain-free but exhibits some type of compensatory movement pattern. A score of three indicates that the participant’s movement pattern is completed as instructed, with no movement compensation noted, and with the movement being pain-free [12]. The total FMS score was calculated by summing the scores of individual elements of the FMS, and therefore the total could range from zero to 21 [12]. The FMS has high interrater reliability [13] and intrarater reliability [14], and the reliability of the FMS within the tactical population has been demonstrated in previous research [13]. This tool was selected due to research in tactical populations linking the movement skills of the FMS and potential for injury, with total FMS scores equal to or below 14 associated with an increased risk of injury [15].

**Figure 1:**
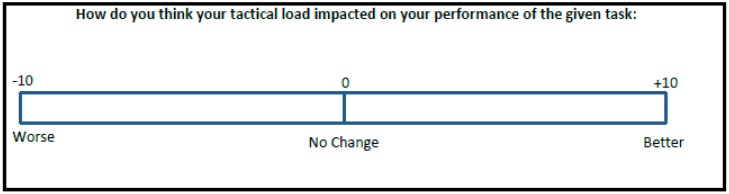
Task-performance impact scoring sheet.

After each of the three movement-dominated tasks (the agility and two simulation tasks), the participants were asked to rate, on a visual analogue scale (Figure 1), the perceived impact of their worn body armour on their ability to complete the respective task. The visual analogue scale was scored from zero to 10 in both a positive and negative direction, along a 200 mm scale. Immediately after each event, officers were asked to mark the scale with a single line. The distance from zero was measured with a ruler and recorded in mm (either positive or negative). The use of a visual analogue scale to determine subjective ratings of task performance impacts with body armour is reported in the literature [16].

**Figure 2:**
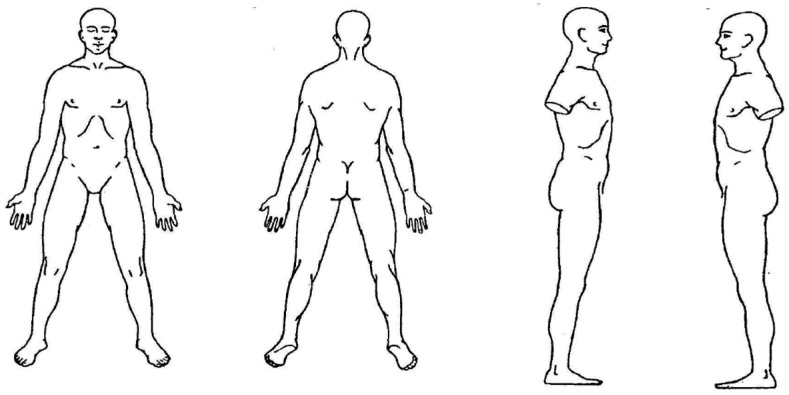
Mannequin sketch to mark any areas of discomfort.

At the completion of the day, when all tasks had been completed and after a minimum of 30 min of sitting, officers were asked to indicate on a mannequin sketch any areas of discomfort they felt from the vests that they were wearing (Figure 2). The use of this approach to investigate subjective feedback on body armour comfort is reported in the literature [16].

### Data Analysis

Data pertaining to postural sway were extracted from the AMTI Bio Analysis software into a Microsoft Excel spreadsheet. In addition to the entire 30-s period pertaining to sway path length, velocity of sway, peak anterior–posterior (AP), and medial–lateral (ML) movements, the data for the first five seconds of sway and the last five seconds of sway were also transferred to another Microsoft Excel spreadsheet. After this data extraction was complete, centre of pressure (COP) coordinates recorded as *x*-axis and *y*-axis COP positions were used to find movement in both the *x* and *y* planes by subtracting each subsequent data point from the previous data point. This revealed any movement of the COP in the plane of interest, which was then input to the Pythagorean theorem (a^2^ + b^2^ = c^2^) to determine the sway path length of the respective individual data point. The total sway path length for the data period of interest (0–5 s or 25–30 s) was then calculated as the sum of the sway path lengths of all data points and divided by five (reflecting the 5 s period of interest, noting velocity = distance/time) to determine the sway velocity for the respective time period.

All data were entered into a data spreadsheet in SPSS (Version 23, International Business Machines, Armonk, NY, USA, 2015) and were cleaned prior to analysis. Demographic data and the performance measure scores for each type of body armour were initially analysed descriptively to derive means and standard deviations to summarise the data. Inferential analyses were then conducted using paired-samples *t*-tests to assess differences between the two types of body armour in associated scores on all performance measures. For comparing individual FMS scores associated with each type of body armour, a Wilcoxon signed rank test analysis was performed, consistent with the ordinal nature of the FMS component data. The overall level of significance was set a priori at 0.05.

## 3. Results

### 3.1. Body Armour Weights

The MBA used in this study on average weighed 6.4 kg, while the LEBA weighed 2.1 kg. There were no differences in weights of the body armour worn by each participant, within each body armour type, since all five of each type (MBA and LEBA) were of the same size. The average (mean) ± SD loaded weights of officers when they were wearing the two different types of vest comprising the body armour can be seen in Table 3.

### 3.2. Postural Sway Results

Table 4 shows the results of the postural sway assessment. There were no significant differences between the two vests with respect to sway in the anterior–posterior direction, medial–lateral direction, the sway path length (SPL), or velocity of sway. For both vests, there were significant differences in both sway path length and velocity between the first five seconds and last five seconds (*p* < 0.05), with these measures increasing by 14.33% and 14.32%, respectively, for the MBA and 11.76% and 11.71%, respectively, for the LEBA.

### 3.3. Vertical Jump Results

While participants were wearing the MBA, the peak force generated during the vertical jump test was 2907.85 ± 1297.37 N on average, while for the LEBA it averaged 3238.96 ± 1568.14 N, representing an 11.39% difference. This difference did not reach statistical significance (*p* = 0.129).

### 3.4. Illinois Agility Test Results

As seen in Table 5, the agility scenario was completed by participants in an average of 21.32 ± 2.64 s while wearing the MBA and 20.65 ± 2.49 s while wearing the LEBA. This represents an average time difference between the two vests of 3.25%, and this was a statistically significant difference, *t*(9) = 4.65, *p* < 0.01. Along with exhibiting a significantly faster time, the lighter LEBA was rated by participants as being significantly less detrimental to performance during the agility task (*t*(9) = −2.819, *p* = 0.02).

### 3.5. Vehicle Exit and 5 m Sprint Results

Time for participants to complete the vehicle exit and 5-m sprint was on average 3.29 ± 0.38 s while wearing the MBA and 3.18 ± 0.46 s while wearing the LEBA, as seen in Table 5. The time to complete was 3.46% slower while wearing the MBA, although this difference was not statistically significant (*p* = 0.197). Despite the lack of a significant difference in time to complete this task, participants again perceived the LEBA to be significantly less detrimental to performance when compared to the MBA during the vehicle exit and 5 m sprint (*t*(9) = −4.912, *p* = 0.001).

### 3.6. Victim Recovery Results

The total time for participants to complete the victim recovery scenario averaged 9.35 ± 1.79 s while wearing the MBA and 9.48 ± 1.65 s while wearing the LEBA. This 1.39% difference was not statistically significant (*p* = 0.529), however participants again perceived the LEBA to be significantly less detrimental to performance in this task than the MBA (*t*(9) = 4.485, *p* = 0.002, Table 5).

### 3.7. Functional Movement Screen (FMS) Results

Total FMS scores averaged 13.40 ± 2.17 and 15.40 ± 1.90, respectively, when the participants were wearing the MBA and LEBA (Table 6). This difference of 14.93% was statistically significant, *t*(9) = 4.743, *p* < 0.001. Inline lunge performance was significantly poorer when wearing the MBA than when wearing the LEBA (*Z* = 2.249, *p* = 0.014).

### 3.8. Subjective Comfort

**Figure 3:**
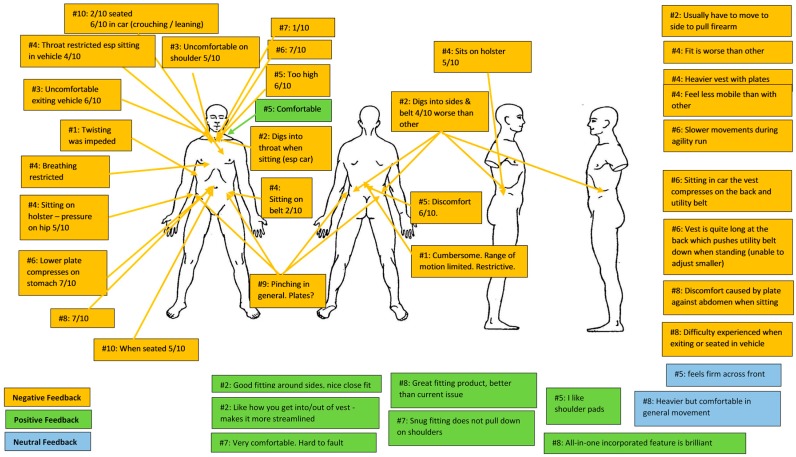
Subjective feedback on military body armour (# signifies participant number; scores out of 10 signify discomfort level).

**Figure 4:**
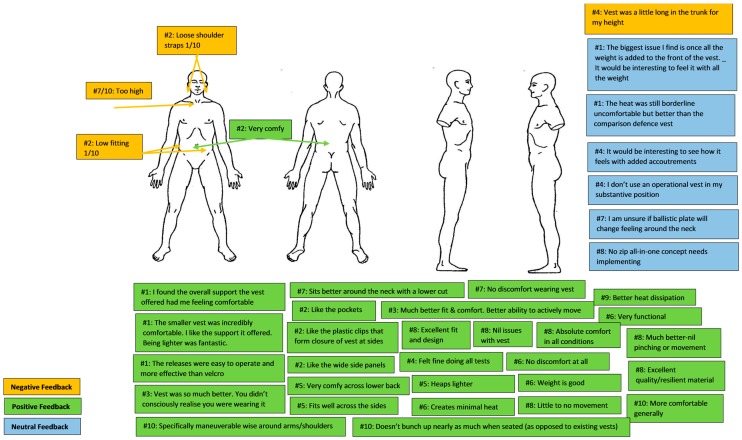
Subjective feedback on law enforcement body armour (# signifies participant number; scores out of 10 signify discomfort level).

In general, the LEBA received notably more positive comments regarding comfort than did the MBA (Figure 3 and Figure 4). The majority of negative comments regarding the MBA referred to: (a) discomfort around the throat and shoulders, especially when sitting (8 comments); (b) the MBA sitting on the belt or holster (6 comments); and (c) compression of the stomach (4 comments). It should be noted that one officer found the MBA to be comfortable around the shoulders, while another stated that they liked the shoulder padding and a third felt that the snug fit meant that the body armour did not pull down on the shoulders. Positive comments regarding the MBA included: (a) the fitting (3 comments) and design features (3 comments).

The negative comments reported by the officers in relation to wearing the LEBA all referred to fit (*n* = 4). The leading positive comments included: (a) comfort / less discomfort (*n* = 10); and (b) fit (*n* = 4). There were also a number of positive comments (*n* = 5) regarding the functionality of the vest (e.g., pockets, wide panels, plastic clips). Although there were only two neutral comments (neither positive nor negative) for the MBA, there were six for the LEBA. Of these six neutral comments, 50% were considerations of how the LEBA may feel if full operational loads and/or plates were added.

## 4. Discussion

The aim of this investigation was to compare the impacts of two different types of body armour on performance of, and comfort during, a variety of measures relating to law enforcement occupational task requirements and simulated policing tasks. When compared with wearing the LEBA, wearing the MBA resulted in significantly slower times to complete an agility task and significantly poorer FMS scores and poorer subjective ratings of performance and comfort across all tasks. Wearing both types of body armour led to an increased velocity of sway and sway path length in the final 5 s of a balance task when compared with the first 5 s, indicating that sway control declined after 30 s of wearing either type of body armour. Body armour type did not significantly affect any other aspect of postural sway or yield any differences in force production during the vertical jump, vehicle exit and 5 m sprint times, or victim recovery times-both types of body armour performed similarly in these respects. The lack of a significant different in the vertical jump may have been influenced by the wide variance in peak forces generated by individual participants during the vertical jump test regardless of the type of vest they were wearing.

Although there was a significant increase in SPL and sway velocity over a 30 s time frame when either type of body armour was worn, there were no significant differences overall, in any measures of sway, between the body armour types. It should be noted, however, that there was a greater increase in SPL and sway velocity in the MBA as opposed to the LEBA, though this did not reach significance. With a larger group size, or with observation over a longer period of time, these findings may have reached statistical significance. Furthermore, although differences were small, when considering repeated exposures, these differences may have significant downstream effects on police populations. Any potential for higher sway velocity effects does raise concern, as load carriage has been associated with an increased risk of trips and falls in some research [17,18] and trips and falls are within the top five injury mechanisms in Australian police populations [7]. Therefore, anything that increases sway velocity may impact on recovery potential following a trip or slip and thus increase the risk of injury.

Times to complete an agility course were significantly slower in the heavier MBA. From the limited research that has been conducted to examine the impacts of load carriage on agility courses, a number of researchers [17,19,20,21,22] have reported increases in time taken to negotiate individual obstacles and obstacle courses to be associated with increases in the weight of the load carried. Therefore, the heavier weight of the MBA is considered to have had a notable impact on the officers’ ability to push up from the ground and to quickly negotiate the course. This observed impact of the heavier MBA in reducing agility is of note given its association with the ability of law-enforcement officers to perform key tasks [11].

There were no significant differences between body armour types in performance of the two simulation tasks (vehicle exit and sprint, and victim recovery). In other research conducted with police populations, with loads (including body armour) ranging from 7.7 kg [2] to 22.8 kg [23], times to complete a vehicle exit and 2.85 m sprint and a 10 m sprint with 10 m victim recovery task were found to be significantly slower than when unloaded. A potential reason for differences in results between the aforementioned studies and the findings in this study may be the larger differences in load weights used in each study, since both of the above-listed studies compared loaded performance with an unloaded condition of only the officer’s body weight. In the current study the loads (vests) were less different in weight. In addition, in the prior studies, physical movement limitations that result from wearing body armour may have contributed to observed differences between loaded and unloaded conditions.

The differences observed in the current study in FMS scores associated with each type of body armour are of note. Research in tactical populations suggests that total scores of below 14 leave participants at a greater risk of injury due to an overall lower quality of movement [15]. In this study, average FMS scores for officers while wearing the MBA was 13.40 points, slightly below the recommended threshold score to reduce injury risk and thus indicating a higher injury risk. Conversely, officers wearing the LEBA were able to achieve average scores of 15.40 points, above the risk threshold score. These results suggest that while wearing the LEBA, officers may be at less risk of musculoskeletal injuries associated with poor movement quality than when wearing the MBA. Furthermore, the average results for the MBA were similar to the mean scores reported by Bock et al. [24] for officers who failed their tactical options assessment (13.32 ± 2.30 points) as opposed to those officers who passed (14.32 ± 1.71 points).

Placing the results of this study in context, previous research in an Australian law enforcement agency has indicated that officers achieve average FMS total scores of 14.93 ± 2.51 points [25]. While the FMS scores observed with the LEBA in the current study were higher than those observed in the aforementioned study, they were nevertheless comparatively lower than those observed in other studies of active duty service members (16.2 ± 2.2) [26], yet similar to the scores found in a population of emergency taskforce police officers (15.1 ± 2.1) [27]. On this basis, the impact of the MBA was such that it lowered the mean FMS movement scores to levels notably below those reported in all other studied tactical population samples.

Overall, LEBA appeared, subjectively, to be the preferred type of body armour by officers when compared with the MBA across all three tasks that were accompanied by a subjective evaluation. Across all three tasks, officers considered the LEBA to enhance ability to complete the task on all occasions, while the MBA was considered to negatively impact on their ability to complete the tasks. When subjective evaluation data from both body armour types were collated (Figure 3 and Figure 4), it was apparent that the major areas of discomfort for body armour were due to fit around the chest/shoulders and hips. However, the LEBA received notably fewer negative comments and substantially more favourable comments than did the MBA. Considering this, modifying the LEBA (e.g., to decrease plate/design torso length) may mitigate some of the concerns raised, but it is possible that making changes would create concerns in other areas and change previous positive or neutral results. Therefore, any modification to LEBA would need to be considered against future possible downstream impacts on comfort in areas other than those that are of current concern. It should be noted that this study examined the performance differences, subjective comfort and perceived effects of these two armour variants. A limitation to this study is therefore that the protective capacity of either of these variants was not investigated. The relative level of protection afforded from each type of body armour would clearly be an important determinant in decision-making before the widespread uptake of any variant for LEOs.

A limitation of this study is the relatively high number of female participants which may be atypical in some police forces. Considering this, the homogeneity of the data, along with each officer serving as their own control, is considered to have mitigated this limitation to some extent.

## 5. Conclusions

The participating officers found LEBA to be more comfortable than the MBA, and they were more positive in their subjective ratings of their own performance during agility and simulation tasks than when wearing the MBA. These subjective responses were supported by significantly faster agility course times when wearing the LEBA than when wearing the MBA, although there were no significant differences in the performance results for the simulation tasks. The MBA was also found to have a significant negative impact on movement quality, as measured by the FMS. The overall performance of officers was reduced to a level previously associated with an increased risk of injury in tactical populations and with failure in a tactical options assessment for LEOs. The results suggest that notable differences between MBA and LEBA exist and they should not necessarily be considered interchangeable, when LEO are considered. On this basis, LEBA specifically designed for law-enforcement as bespoke equipment is vital if the health and safety of officers is to be ensured.

## Figures and Tables

**Table 1:** Demographic details (mean ± SD) of the participants.

	Males	Females	All
**Number**	4	6	10
**Height (cm)**	182.15 ± 6.98	167.97 ± 3.67	173.64 ± 8.80
**Weight (kg)**	85.55 ± 9.96	65.30 ± 10.57	73.40 ± 15.00

**Table 2:** Schedule for each day of testing.

Time	Activity
**0830**	Briefing and vest allocation
	Initial anthropometric measures *
**0900**	Postural sway measures and counter movement jump
**0940**	Illinois Agility Test
**1010**	Vehicle exit and 5 m sprint
**1030**	10 m sprint to simulated victim and 10 m recovery drag
**1100**	Functional Movement Screen
**1130**	Lunch (wearing allocated vests)
**1200**	Subjective assessments

* Participants’ height and unloaded weight measurements were taken on the first day only.

**Table 3:** Loaded weights (mean ± SD; kg) of officers, by gender and type of body armour.

	Males	Females	All
**Vest military body armour (MBA)**	92.18 ± 9.98	71.58 ± 10.88	79.82 ± 14.56
**Vest law-enforcement body armour (LEBA)**	87.68 ± 10.02	67.32 ± 10.49	75.46 ± 14.33

**Table 4:** Postural sway results (mean ± SD) between the different body armour types.

Time	Measure	Vest MBA	Vest LEBA
**First 5 s**	Peak AP (cm)	1.34 ± 0.33	1.18 ± 0.30
	Peak ML (cm)	1.19 ± 0.32	1.25 ± 0.35
	SPL (cm)	20.93 ± 3.20	21.35 ± 3.20
	Vel (cm/s)	4.19 ± 0.58	4.27 ± 0.64
**Last 5 s**	Peak AP (cm)	1.37 ± 0.59	1.19 ± 0.66
	Peak ML (cm)	1.12 ± 0.38	1.23 ± 0.46
	SPL (cm)	23.93 ± 3.77 *	23.86 ± 2.66 *
	Velocity (cm/s)	4.79 ± 0.75 *	4.77 ± 0.53 *
**Total**	Peak AP (cm)	2.68 ± 1.07	2.52 ± 0.93
	Peak ML (cm)	2.19 ± 0.63	2.15 ± 0.69
	SPL (cm)	122.45 ± 15.85	126.17 ± 19.99
	Velocity (cm/s)	4.08 ± 0.53	4.21 ± 0.67

* Significantly greater than the first 5 s for the same vest (*p* < 0.05). AP, anterior–posterior; ML, medial-lateral; SPL, sway-path length

**Table 5:** Task Performance and subjective results (mean ± SD) for the different body armour types.

Activity	MBA	LEBA
*Agility*		
Subjective Rating	−2.78 ± 3.20	2.45 ± 4.40 *
Time (s)	21.32 ± 2.64	20.65 ± 2.49 ^†^
*Vehicle exit and 5 m sprint*		
Subjective Rating	−3.07 ± 4.17	3.68 ± 3.54 *
Time (s)	3.29 ± 0.38	3.18 ± 0.46 ^†^
*10 m sprint and 10 m victim recovery*		
Subjective Rating	−0.29 ± 2.62	3.76 ± 3.21 *
Time (s)	9.35 ± 1.79	9.48 ± 1.65

* Significantly better rating than Military Body Armour (MBA) (*p* < 0.05); ^†^ significantly quicker than MBA (*p* < 0.01).

**Table 6:** Functional movement screen (FMS) results (mean ± SD) by body armour type and FMS movement element, and overall.

	MBA	LEBA
Deep squat	2.20 ± 0.79	2.50 ± 0.71
Hurdle step	1.80 ± 0.63	1.80 ± 0.79
Inline lunge	2.10 ± 0.74	2.70 ± 0.48 *
Shoulder mobility	1.40 ± 0.52	1.90 ± 0.57 ^‡^
Active straight leg raise	1.80 ± 0.79	2.30 ± 0.67
Trunk stability pushup	2.30 ± 0.82	2.60 ± 0.52
Rotary stability	1.80 ± 0.79	1.60 ± 0.84
Total	13.40 ± 2.17	15.40 ± 1.90 *

* significantly greater than with MBA vest (*p* < 0.05); ^‡^ *p* = 0.059.
